# Impact of Nutrition Education on Various Health-Related Components of Hemodialysis Patients: A Systematic Review

**DOI:** 10.3390/healthcare12121197

**Published:** 2024-06-14

**Authors:** Marouane Ouirdani, Amal Boutib, Asmaa Azizi, Samia Chergaoui, El Madani Saad, Abderraouf Hilali, Abdelghafour Marfak, Ibtissam Youlyouz-Marfak

**Affiliations:** 1 Laboratory of Health Sciences and Technologies, Higher Institute of Health Sciences, Hassan First University of Settat, Settat 26000, Morocco; boutibamal@gmail.com (A.B.); a.azizi@uhp.ac.ma (A.A.); samia.chergaoui@uhp.ac.ma (S.C.); saad.elmadani@uhp.ac.ma (E.M.S.); abderraouf.hilali@uhp.ac.ma (A.H.); ibtissam.marfak@uhp.ac.ma (I.Y.-M.); 2 National School of Public Health, Ministry of Health and Social Protection, Rabat 6329, Morocco; ab.marfak@gmail.com; 3 Pole of Health, Euro-Mediterranean University of Fez (UEMF), Fez 51, Morocco

**Keywords:** nutrition education, hemodialysis, review

## Abstract

This study aimed to identify the impact of nutrition education on various health-related components of hemodialysis patients. A systematic review was conducted according to the PRISMA guidelines. Relevant literature published between 2013 and 2023 was identified across two databases (PubMed and Science Direct). The protocol was registered in PROSPERO (CRD42023460681). Two independent reviewers retrieved the data, and 41 studies were selected. Nine components related to the impact of nutrition education in hemodialysis patients were identified. Each component was clarified by mentioning each study and its results. This study enabled us to characterize the various components of the impact of nutritional education in hemodialysis patients, namely biological markers, quality of life, cost of care, adherence to dietary recommendations, knowledge, malnutrition inflammation, dietary intake, weight change, and behavior change. This systematic review enables healthcare providers to assess the impact of nutritional education on hemodialysis patients. Also, it gives professionals an exact idea of the impact of nutrition education on hemodialysis patients, with knowledge of new methods using behavior change theories and innovative technological tools.

## 1. Introduction

Statistics have revealed an increase in non-communicable diseases responsible for an estimated 41 million people dying annually, constituting 74% of all deaths worldwide [1]. In the 21st century, chronic kidney disease (CKD) has emerged as a leading cause of death and disability. As risk factors like obesity and diabetes mellitus have increased, CKD patients have also increased in number. By 2017, 843.6 million people were estimated to be affected globally. Thus, increased efforts should be made to improve the prevention and treatment of chronic kidney disease due to the large number of affected individuals and the serious consequences of the condition [2].

According to the USRDS annual data report, the average annual change in the incidence of ESRD treated by country or region demonstrates an increase in most countries participating in the survey. Hemodialysis emerges as the primary treatment modality for end-stage renal failure worldwide [3].

Hemodialysis patients encounter numerous challenges, encompassing the management of fluid and phosphate intake [4], anemia, cardiovascular problems, protein-calorie malnutrition, infections, renal osteodystrophy, and interdialytic complications [5]. In this regard, several alternatives are used to manage chronic renal failure, especially among hemodialysis patients, including health education. This concept is defined by the World Health Organization (WHO) as “health education compris[ing] consciously constructed opportunities for learning involving some form of communication designed to improve health literacy, including improving knowledge and developing life skills which are conducive to individual and community health” [6]. In this respect, the United States National Commission for Health Education Credentialing (NCHEC) has determined the health educator’s seven main duties as: “assessing individual and community needs for health education, planning effective health-education programs, implementing health-education programs, evaluating the effectiveness of health-education programs, communicating health and health-education needs, concerns and resources, coordinating the provision of health-education services and acting as resource people in health education” [7].

Many health-education interventions for hemodialysis patients have been implemented, covering various themes such as physical exercise, medication adherence, and nutrition. Systematic reviews have been conducted on the impact of education in general [8] and specific themes [9]. However, to date, no review has specifically examined the impact of nutrition education on hemodialysis patients.

The specific objective of this review was to assess the impact of nutrition education on patients undergoing hemodialysis. This systematic review aimed to address the following questions:

- Can nutrition education improve outcomes for patients undergoing hemodialysis?
- Which health-related outcomes can be modified by nutrition education?

## 2. Materials and Methods

### 2.1. Study Selection and Search Strategy

We conducted a systematic literature search covering the period from 1 January 2013 to 8 September 2023. The search terms were organized into three queries:(1) “health education AND hemodialysis”; (2) “patient education AND hemodialysis”; (3) “lifestyle AND hemodialysis”. Additionally, for the period from 1 January 2013 to the end of 2023, we included the queries: (1) “health education AND haemodialysis”; (2) “patient education AND haemodialysis”; (3) “Self care AND haemodialysis” in two databases (PubMed and ScienceDirect). The chosen timeframe spanned 11 years to ensure the inclusion of as many recent studies as possible. The search strategy was agreed upon by all the authors, who brought diverse expertise in nursing, public health, and biology.

**Figure 1:**
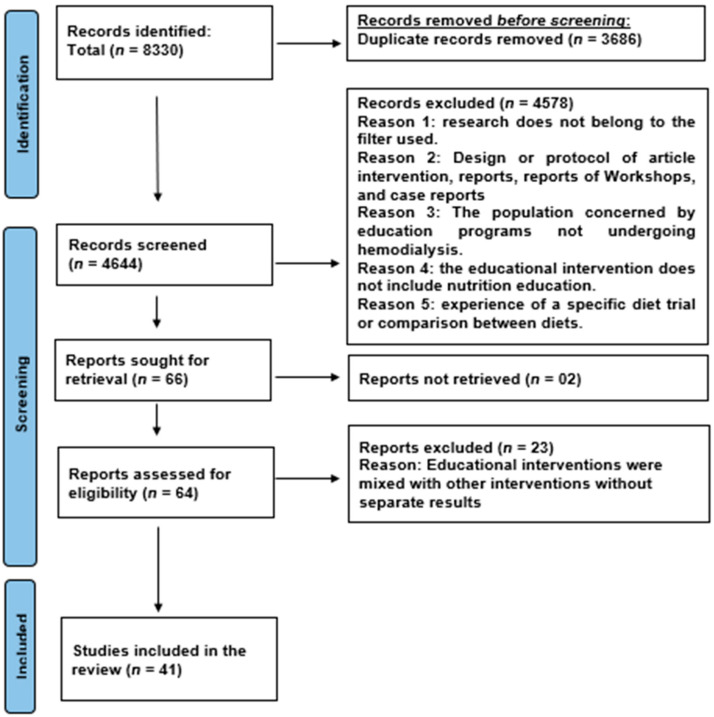
PRISMA flow chart of the selection process.

The flow diagram (Figure 1) illustrates the search strategy, detailing all study selection and elimination stages.

This review is registered in PROSPERO with ID CRD42023460681 and respects the Preferred Reporting Items for Systematic Reviews and Meta-Analyses (PRISMA) 2020 guidelines [10] (See PRISMA 2020 checklist in supplementary materials).

### 2.2. Inclusion and Exclusion Criteria

Studies, regardless of the language of publication and the study design (i.e., quantitative or qualitative), were excluded if they exhibited the following characteristics:

- Research does not belong to the filter used (research articles, clinical trials, randomized controlled trials).
- Design or protocol of article intervention, reports, reports of workshops, and case reports.
- Population concerned by the education programs not undergoing hemodialysis.
- The educational intervention does not include nutrition education.
- Experience of a specific diet trial or comparison between diets.

In the second stage of selection, the remaining articles were downloaded and reviewed in their entirety. Studies were excluded if educational interventions were mixed with other interventions without separate results.

### 2.3. Data Extraction and Quality Assessment

Studies were independently selected and reviewed by two of the authors (O.M. and B.A.) based on predefined eligibility criteria. RIS files downloaded according to the 6 requests were transferred to the Rayyan platform, a recommended screening system for literature reviews and systematic reviews [11], to eliminate items not meeting the first initial screening criteria. Complete data extraction was conducted when the intervention focused solely on nutrition education. However, in cases where interventions were multidisciplinary and outcomes were separated, only the data related to the nutrition component were extracted, ensuring that they were not influenced by other interventions.

For example, in the case of interventions involving nutrition education and phosphate binder education, data on serum phosphate levels were excluded, as it was unclear which intervention influenced this parameter. However, we did include interventions containing brief education on medication, or assisted by personalized food plans as part of nutrition education. To address the research questions, the results of the selected studies were categorized into several sections that were aligned with the studies’ objectives. The outcomes were either compared between pre- and post-intervention within the same group or between intervention and control groups (with or without randomization).

Two authors (M.O. and A.B.) participated in assessing the methodological quality of the included articles using two tools: the JBI Critical Appraisal Checklist for Case Series (10 items) and The Quantitative Study Quality Assessment Tool Rating (31 items). Details of the quality assessment are provided in Supplementary Materials.

## 3. Results

The systematic search yielded 8330 articles (Figure 1), of which 3686 duplicates were removed. Following title and abstract screening, 66 articles were selected for full review, of which 2 full texts were not found, although it is highly likely that they would not have been included, having read the abstract. After a full-text review and quality assessment, 41 articles were ultimately included for analysis.

Among the 41 included studies (Table 1), a significant proportion were conducted in Asia (*n* = 23), followed by South America (*n* = 6), North America (*n* = 5), Europe (*n* = 3), Africa (*n* = 2), and Oceania (*n* = 1). Notably, one study was conducted across four different continents. Regarding study design, 51.2% of the studies were randomized controlled trials. Most studies (75.6%) were conducted in hemodialysis units, dialysis centers, nephrological units, and clinics, and 22% were carried out in hospitals or teaching hospitals. The total sample size of patients included in the final analysis was 5243. In terms of publication dates, 43.9%of the studies were published between 2013 and 2018, with the remaining studies published up to 2023. Table 2 provides a detailed overview of each study’s aims and relevant results.

### 3.1. Areas of Action Studied, with Results Achieved in Each Study: (All Reported Changes Are Statistically Significant)

#### 3.1.1. Impact of Nutrition Education on Hemodialysis Patient Knowledge

Patients should be able to recognize the importance of the composition of certain dietary ratios and the implementation of healthy cooking methods to achieve optimal efficacy.

Nutritional education has proven effective in improving hemodialysis patients’ knowledge in various areas, as demonstrated by the results of several studies in this review. Significant improvement was observed in nutritional and dietary knowledge scores [16,21,24,25,26,30,33,51], as well as knowledge of fluid control [23,24].

The clinical, behavioral, and biological manifestations of these educational interventions will be covered in the following sections.

#### 3.1.2. Impact of Nutrition Education on Hemodialysis Patient Behavior/Attitude Change

With the emergence of new diseases associated with longevity and lifestyle factors, the biomedical model has been superseded by models linked to health psychology. These models enable the description of behavioral changes and the development of targeted prevention interventions across primary to tertiary [53]. According to the results of certain studies reviewed, dietary behavior has shown significant movement when associated with nutritional education [18,21,22,23,32,33].

#### 3.1.3. Effect of Nutrition Education on Weight Change

One of the major challenges encountered by hemodialysis patients is interdialytic weight gain, which serves as an indicator of higher pre-dialytic blood pressure, nutrition, and survival in hemodialysis (HD) patients [54]. In this review, nutritional education, particularly concerning fluid restriction and control, has demonstrated satisfactory outcomes in reducing IDWG [13,19,23,43] and achieving the ideal percentage of weight loss [34]. Furthermore, when combining nutrition education with cognitive behavioral therapy, positive results were observed in terms of reducing IDWG [44].

#### 3.1.4. Effect of Nutrition Education on Malnutrition Inflammation Status

Inflammation and protein-energy malnutrition (PEM) are common and often concurrent conditions among patients undergoing hemodialysis [55]. This review demonstrated that the implementation of nutritional education programs had a beneficial effect on the malnutrition inflammation score [22].

#### 3.1.5. Effect of Nutrition Education on Cost of Care

People afflicted with chronic kidney disease are at a greater risk of fatal complications, leading to elevated costs for both patients and healthcare institutions. However, following nutritional interventions has been associated with a lower length of hospital stay [31] and reduced direct costs [35].

#### 3.1.6. Effect of Nutrition Education on Dietary Intake

Due to the specific nature of the disease and renal function deterioration, certain elements are no longer effectively eliminated by the body, posing a risk of harm to certain organs at high concentrations. Consequently, there is a need to limit their intake. However, adherence to certain dietary restrictions can lead to undernutrition. In this regard, nutritional education has played a crucial role in addressing this imbalance. Analysis of selected studies has demonstrated that nutritional education has led to an increase in protein intake [12,17,18,28], P/Protein ratio [21], phosphorus intake [12,18], Energy intake [18], lipids intake [18], mono and saturated fat intake [18], copper intake [18], vitamin C intake [18], linolenic acid intake [18], cholesterol intake [18], iron intake [18], potassium intake [18], along with a decrease in sodium intake [14,19,37,41], carbohydrate intake [18,28], polyunsaturated fat intake [18], zinc intake [18], potassium intake [19,28], phosphorus intake [19,22,28], protein intake [19,31].

#### 3.1.7. Effect of Nutrition Education on Adherence to Dietary Recommendations

The studies included in this review have demonstrated an increase in adherence to fluid restriction [13,15,39,40,43,44,49], potassium and phosphate intake [13], dietary restriction [15,39,40,44,49], specific cooking methods [28], vegetable preferences [28], sodium intake adherence [13], and reduction in the frequency of non-adherence to the diet [23], frequency of non-adherence to fluid restriction [23], the score of non-adherence to sweets high in P (phosphorus) [27], and starch high in P [27].

#### 3.1.8. Effect of Nutrition Education on the Quality of Life of Hemodialysis Patients

Quality of life is a crucial health outcome and represents the ultimate goal of all health interventions [56]. Nutrition education has demonstrated this in two studies included in this review by increasing the quality-of-life score [26,29].

#### 3.1.9. Effect of Nutrition Education on Biological Markers

Biological markers are biological molecules that indicate a normal or aberrant process and can be discovered in tissues, blood, or other bodily fluids [57]. Results from the reviewed studies revealed significant changes in several markers following nutritional education through the increase in serum protein [16], serum potassium [16], serum calcium [16,40], C-reactive protein [16], hemoglobin [18,40], hematocrit [18], protein [18], globulin [18], saturation of transferrin [18], serum PTH [36], IRON [40], albumin [40,47], and decrease among patients with high parathyroid hormone [12], serum phosphorus [12,13,18,21,22,28,29,31,32,40,47,50], calcium [18], creatinine [18,40], potassium [13,18,28,29,40,50] blood urea nitrogen [31], pre- and post-dialysis urea [18], albumin [18], calcium [20], Ca/P product [20], Ca*P product [27], hemoglobin [20], % of patients with potassium over 5.5 mEq/L [20], % of patients with phosphorus over 5.5 mg/DL [20], phosphorus less than 3.5 mg/DL [20], sodium [29,40], magnesium [29], BUN [40,51], median percent time INR less than 1.5 [46].

## 4. Discussion

After extracting and analyzing the results of the selected studies, the contribution of nutrition education has been limited to nine general themes (knowledge, patient behavior/attitude change, weight change, malnutrition inflammation status, dietary intake, adherence to dietary recommendations, quality of life of hemodialysis patients, and biological markers).

Compared to pre-nutritional education, participants showed increased scores in both the knowledge component of fluid control and dietary restrictions/limitations. Similar findings were reported in a study, which concluded that diabetes patients’ knowledge and practices about nutrition were improved by nutritional and eating education. This best practice empowered them to control their blood glucose levels effectively [58].

In terms of cost of care, nutrition education was less expensive than other interventions, which is in line with the results of a nutrition education program for reducing childhood obesity, whose findings show that the intervention seems to be both effective and cost-effective [59].

A positive change has also been observed in the interdialytic weight gain (IDWG) level, with reductions achieved and satisfactory results obtained. In comparison to another study, dietary advice and food fortification led to weight gain and improved outcomes during and after the intervention period in nutritionally vulnerable outpatients with chronic obstructive pulmonary disease [60]. These findings demonstrate that targeted weight goals can be achieved through nutrition education. Furthermore, nutritional education has kept the level of inflammatory malnutrition stable and has increased patients’ quality of life. This finding aligns with results indicating that a nutrition education program significantly improved the quality of life of adults living with HIV [61].

In several studies included in this review, regression was recorded in serum phosphorus, potassium, sodium, and creatinine, as well as an increase in serum protein. Additionally, the effectiveness of nutritional education in improving biological markers was also observed in diabetic patients by reducing HB1AC and BMI [62].

Compared with other systematic reviews, a review of 43 studies found that school student’s health education improved their knowledge, attitudes, perceptions, and behaviors related to physical activity and nutrition [63]. Another review comprising 6 studies concluded that nutrition education among Indigenous Australians contributed to the reduction of anthropometric factors and biochemical risks associated with chronic diseases [64].

According to the findings of another analysis, teaching programs focused on physical activity and nutrition may effectively assist African Americans in achieving clinically meaningful results [65]. Older individuals who modify their diets through dietetic education or have access to more healthful meal services could be able to obtain better-quality food [66].

The studies in our review were subject to certain limitations. This included insufficient results to draw any conclusions [12], challenges in generalizing the findings [13,15,27,35,48,50,51], language difficulties among patients [13], a lack of detail on the effect of the intervention elements [13], the possibility of contamination of information between patient groups [14,19,34,44], uncontrolled variables [14,21,22,26,29,31,37], short duration of studies, interventions, or follow-up [15,19,27,30,35,41,51], the patients already being familiar with educational content [17], small sample size [29,30,37,41,46,49,52], self-selection bias [52], not all information being analyzed [20,48], subjective results linked to patient declarations [23], inaccurate assessment tools [42], possible Hawthorne effect [30], interventions conducted in a single center [30,41], and the possibility of social desirability effect bias [39,52].

Another limitation of this review is the potential for expanding the number of search databases, which could lead to the inclusion of additional studies.

The provision of care for hemodialysis patients represents a substantial burden, affecting patients, families, and healthcare institutions alike. In this context, health education has demonstrated considerable efficacy across multiple domains.

Nevertheless, patient education in hemodialysis encounters various obstacles, particularly related to commitment and adherence to restrictions. Among these challenges, the psychological state of patients represents a significant hurdle, with a notably high prevalence of depression observed [67]. Depression has been linked to suboptimal adherence to medication regimens [68,69], as well as dietary non-adherence [70]. In this regard, further studies using psychological models need to be conducted to address the issue of depression in hemodialysis patients, particularly those from lower socio-economic backgrounds or underdeveloped countries.

## 5. Conclusions

Our study empirically confirmed the positive impact of nutrition education on hemodialysis patients, addressing several critical aspects. These findings underscore the importance of healthcare providers incorporating nutrition education into the standard treatment for hemodialysis patients.

While the results of this review underscore the significance of nutrition education for hemodialysis patients, it is essential to recognize that comprehensive care extends beyond nutrition alone. Healthcare providers must adopt a holistic approach to achieve optimal outcomes. Furthermore, this review provides valuable insights into the precise impact of nutrition education on hemodialysis patients, incorporating contemporary methods rooted in behavior change theories and leveraging innovative technological tools.

In this regard, healthcare providers are tasked with implementing nutritional educational interventions tailored to optimize benefits for individuals affected by this condition, taking into account the availability of educational resources within their respective healthcare establishments.

## Figures and Tables

**Table 1:** Characteristics of included studies (*n* = 41).

**SETTINGS**	**N°**		**%**	
Hemodialysis Units/Dialysis Centers/Nephrological Units and Clinics	31		75.6%	
Hospital; Teaching Hospital	9		22%	
Not Specified	1		2.4%	
**DATABASES**	**N°**		**%**	
PubMed	28		68.3%	
Science Direct	3		7.3%	
PubMed + Science Direct	10		24.4%	
**STUDY DESIGN**	**N°**		**%**	
Randomized Controlled Trial	21		51.2%	
Clinical Trial/Experimental Studies	08		19.5%	
Observational study	01		2.4%	
Randomized study	08		19.5%	
Non-randomized trial	2		4.9%	
quasi-experimental study	1		2.4%	
**USE OF THEORETICAL OR CONCEPTUAL FRAMEWORK**	**Yes**		**No**	
	**N°**	**%**	**N°**	**%**
	31	75.6%	10	24.4%
**LANGUAGE**	**N°**		**%**	
English	40		97.6%	
French	01		2.4%	
**EDUCATOR PROFILE**	**N°**		**%**	
Nephrologists/Dieticians/Physicians/Medical Workers/specialists in psychiatric/ Registered pharmacists/Nurses	28		68.3%	
Not Specified	13		31.7%	
**NUTRITION SOFTWARE**	**N°**		**% from all studies**	
The computer software Nutritionist Pro™ (Axxya Systems LLC, Stafford, TX, USA, https://nutritionistpro.com/)	01		2.4%	
Xyris Software Foodworks, version 9	01		2.4%	
DietPro software, version 5.8	01		2.4%	
The Balancelog (Microlife; Golden, CO, USA, https://metabolicratetest.com/) Software Program	01		2.4%	

**Table 2:** Objectives and relevant results of selected studies (n = 41).

Author, Year, Reference	Title	Country	AIMS	Sample Size of Hemodialysis Patients (Completing Studies)	Nutritional Intervention Data Collection Tool	Theoretical or Conceptual Framework Used	Relevant Results
A. L. Stieber and al., 2015 [12]	Using a Web-Based Nutrition Algorithm in Hemodialysis Patients	- United States of America - New Zealand - Ireland - Australia - Brazil	- The objective of the evaluation is to ascertain whether the algorithm can identify patients who are at risk, whether the risk assessment is valid, and whether the algorithm’s clinical decision support system produces logical reasoning evidence. - The algorithm’s capacity to collect follow-up data, connect it to earlier visits, and show how patient outcome measures have changed over time was used to assess the second goal.	100	The web-based nutrition algorithm.	- The International Dietetics and Nutrition Terminology (IDNT) Reference Manual	- Patients in the insufficient protein group showed a significant increase in protein intake. - The average intake of phosphorus among individuals classified as belonging to the high phosphorus group rose by 157.27 mg/day. - Serum phosphorus decreased on average by 0.91 mg/dL among patients diagnosed in the high phosphorus group.
Griva et al., 2018 [13]	Hemodialysis Self-management Intervention Randomized Trial (HED-SMART): A Practical Low-Intensity Intervention to Improve Adherence and Clinical Markers in Patients Receiving Hemodialysis	Singapore	To compare Hemodialysis Self-management Intervention versus standard medical care.	193	- The Renal Adherence Behavior Questionnaire - The self-monitoring and insight, constructive attitudes and approaches, skill and technique acquisition, and health service navigation subscales—Self-Efficacy for Managing Chronic Disease questionnaire.	- The Healthy Eating for People on Dialysis educational booklet - NKF-KDOQI (National Kidney Foundation–Kidney Disease Outcomes Quality Initiative) recommendations were used as target ranges - Social-cognitive theory	- The score of adherence to fluid restriction in the HED smart group increased between baseline and 3 times. - Concerning adherence to potassium and phosphorus intake, the HED smart group compared to usual care, had significantly the highest score in T2, T3, and T4. - Regarding adherence to sodium intake, a significant difference was in favor of the HED smart group in T2 and T4. - Significantly lower phosphate concentration difference was identified in favor of the HED smart group compared to usual care at both T3 and T4.
De Freitas et al., 2020 [14]	Effects of dietary counseling on sodium restriction in patients with chronic kidney disease on hemodialysis: A randomized clinical trial	Brazil	The purpose of this study was to assess how dietary counseling affected patients receiving HD treatment concerning sodium restriction and how it related to clinical, dietary, and quality-of-life parameters.	62	- Food frequency questionnaire specific for sodium (FFQ-So) - Patient’s medical records - Brazilian version of the Kidney Disease and Quality-of-Life Short Form.	Not mentioned	The intervention group’s total sodium intake significantly decreased during the three months after dietary recommendations.
Dsouza et al., 2023 [15]	Effect of Educational Intervention on Knowledge and Level of Adherence among Hemodialysis Patients: A Randomized Controlled Trial	India	The study’s objectives were to characterize the relationship and evaluate the effect of an educational intervention on hemodialysis (HD) patients’ knowledge and adherence to their treatment plans and the relationship between them.	160	- A questionnaire on sociodemographic and clinical characteristics - The ESRD-AQ explores all dimensions of HD patient adherence.	- Inputs from DOQI guidelines and in-depth interviews with experts of inter-disciplines - Nephrologist, dietary, pharmacy, and physical therapy	- Adherence to fluid restriction increased significantly in the intervention group. - In terms of adherence to dietary restriction, the intervention group showed an increase between baseline and post-intervention.
Hernández Morante et al., 2014 [16]	Effectiveness of a Nutrition Education Program for the Prevention and Treatment of Malnutrition in End-Stage Renal Disease.	Spain	The study’s objectives were to treat and prevent malnutrition in these patients by implementing a rigorous 4-month nutrition education program and assessing its efficacy.	87	- Self-reported questionnaire.	- The recommendations contained in the International European Guidelines - National Kidney Foundation guidelines	- The NEP group significantly increased its score on nutritional knowledge at the end of the study. - The total serum protein content of the NEP group after 2 months of treatment was significantly higher than that of the OS group.
St-Jules et al., 2021 [17]	Feasibility and Acceptability of mHealth Interventions for Managing Hyperphosphatemia in Patients Undergoing Hemodialysis.	USA	To assess the viability and acceptability of phosphorus management initiatives in mobile health (mHealth) among patients receiving hemodialysis (HD).	40	- Medical records and questionnaires.	- Social cognitive theory-based behavioral	- From the start of the study to the third month, reported protein consumption decreased in the Education group and increased significantly in the “Monitoring” and “Combined” groups. This finding remained significant for up to 6 months between the Education and Monitoring groups.
Vaz De Melo Ribeiro et al., 2020 [18]	Effect of a Nutritional Intervention, Based on Transtheoretical Model, on Metabolic Markers and Food Consumption of Individuals Undergoing Hemodialysis	Brazil	To assess how a nutritional intervention based on the transtheoretical model affected the dietary intake and metabolic markers of patients receiving hemodialysis (HD).	83	- Semi-structured interview, with the application of a sociodemographic and health questionnaire. - Medical records.	- The behavioral change stage model, also known as the transtheoretical model - Recommendation proposed by the National Kidney Foundation - Kidney Disease Outcomes Quality Initiative. - Fouque’s recommendations for Total fibers and micronutrients.	- Knowledge about phosphorus showed a significant progression between pre and post-intervention. - A significant increase in daily Food consumption was marked in Energy, protein, lipids, Monounsaturated fat, saturated fat, Copper, Vitamin C. iron, phosphorus, potassium, and a significant decrease in carbohydrates, polyunsaturated fat, and Zinc.
Dawson et al., 2021 [19]	A Text Messaging Intervention for Dietary Behaviors for People Receiving Maintenance Hemodialysis: A Feasibility Study of KIDNEYTEXT	Australia	To evaluate the feasibility of a mobile phone text-message intervention to assess the viability of a text-message intervention on a mobile device to enhance eating habits in hemodialysis patients to improve dietary behavior in patients receiving hemodialysis.	115	- Interviews - The Healthy Eating Index - The EuroQol 5 Dimension - Medical records	- Dietary guideline recommendations	- The intervention group had a significant improvement in IDWG compared to the usual care group. - Mean sodium intake decreased more in the intervention group than in the usual care group.
Valente et al., 2022 [20]	Dietary Advice in Hemodialysis Patients: Impact of a Telehealth Approach During the COVID-19 Pandemic	Portugal	To evaluate the impact of nutritional interventions provided via telehealth on clinical and nutritional parameters in HD patients undergoing maintenance during the coronavirus outbreak-caused confinement.	156	- The phone calls.	Not mentioned	- Regression was observed in the percentage of patients with potassium over 5.5 mEq/L, patients with phosphorus over 5.5 mg/dL, and patients with phosphorus less than 3.5 mg/dL.
Karavetian et al., 2015 [21]	Effect of behavioral stage-based nutrition education on management of osteodystrophy among hemodialysis patients, Lebanon	Lebanon	Examine the impact of thorough nutrition instruction provided by trained, dedicated dietitians on the management of osteodystrophy in hemodialysis patients.	394	- Semi-structured qualitative interviews - Knowledge questionnaire - Medical record.	- The transtheoretical model (TTM) or the stages of behavioral change model - The KDOQI nutritional guidelines for HD patients - Recommendations by the Academy of Nutrition and Dietetics (ADA-EAL)	- Only the DD group had significantly improved his knowledge to an acceptable level at T1 and maintained it at T2. - The DD group was the only one to experience significant regression in serum P.
Rizk et al., 2017 [22]	Effect of stage-based education provided by dedicated dietitians on hyperphosphataemic hemodialysis patients: results from the Nutrition Education for Management of Osteodystrophy randomized controlled trial	Lebanon	Sought to evaluate and compare the efficacy of intensive, stage-based nutrition education given by qualified dietitians to hyperphosphataemia management in patients receiving hemodialysis who were hyperphosphatemic.	177	- The malnutrition inflammation score - Medical records.	- The transtheoretical model of behavioral change - Kidney Disease Outcomes Quality Initiative guidelines	- Only the DD group maintained a stable score at the end of the study concerning the score of malnutrition inflammation; on the contrary, the average THD and EP group scores showed a significant increase, which indicates a deterioration in their nutritional status.
Nadri et al., 2020 [23]	Impact de l’éducation du patient en hémodialyse sur le respect des mesures diététiques et sur la restriction aux liquids.	Morocco	To assess the impact of implementing a program that educates on following dietary guidelines and limiting liquid intake.	50	- Sociodemographic questionnaire - Dialysis diet and fluid non-adherence questionnaire (DDFQ) questionnaire - Fluid Control in Hemodialysis Patients Scale (FCHPS) questionnaire.	Not mentioned	- After the introduction of the educational program, there was a significant difference (*p* = 0.02) in favor of the intervention group in interdialytic weight gain.
Fadlalmola et al., 2020 [24]	Impact of an educational program on knowledge and quality of life among hemodialysis patients in Khartoum state	Sudan	To assess how well an educational program has affected the knowledge and standard of living of hemodialysis patients in the state of Khartoum.	100	- Interviewing questionnaires - Ferran and Powers Quality-of-Life Index of dialysis.	Not mentioned	Knowledge of dietary restrictions/limitations, liquid restrictions, and authorized foods increased between the pre-test and the post-test.
Yin et al., 2021 [25]	Implementation and effectiveness of an intensive education program on phosphate control among hemodialysis patients: a non-randomized, single-arm, single-center trial.	China	To create a comprehensive education program for hemodialysis patients that focuses on phosphate control and assess its efficacy.	346	- Questionnaire to collect the participants’ sociodemographic and clinical data - The Patient Questionnaire on Phosphate Control Knowledge.	- KDIGO guidelines, - The Chinese CKD-MBD guidelines	Knowledge of diet has significantly increased (*p* < 0.001) from before the program to 6 months after the program.
Ebrahimi et al., 2016 [26]	Influence of nutritional education on hemodialysis patients’ knowledge and quality of life.	Iran	To develop and evaluate an all-inclusive education program with a phosphate control focus for hemodialysis patients.	99	- Demographic questionnaire - Questionnaire regarding subjects’ dietary status - The standard questionnaire to assess QOL for ESRD patients.	Not mentioned	- The experimental group showed a significant increase in mean knowledge score after the intervention, while the control group showed no significant difference.
Karavetian et al., 2013 [27]	NUTRITIONAL EDUCATION FOR THE MANAGEMENT OF OSTEODYSTROPHY (NEMO) IN PATIENTS ON HAEMODIALYSIS: A RANDOMISED CONTROLLED TRIAL.	Lebanon	To investigate how hemodialysis patients’ adherence to dietary management of hyperphosphatemia is affected by self-management dietary counseling (SMDC).	87	- Medical charts - A patient knowledge (PK) questionnaire - A patient’s dietary non-adherence (PDnA) to a phosphate-restricted diet questionnaire.	- Self-management counseling (using cognitive behavioral therapy)	- The intervention group was the only one to show a change with a significant increase in the level of knowledge, while other groups had no significant change - Only the intervention group has a significant improvement in overall patient dietary non-adherence.
Rahman et al., 2022 [28]	Provision of renal-specific nutrition knowledge for changing dietary practice in Bangladeshi hemodialysis patients.	Bangladesh	To assess how nutrition education has affected Bangladeshi dialysis patients’ dietary habits.	50	- 10 items multiple choice questions - The dietary reports were gathered using the 3-day dietary recall (3DDR) approach. - Medical records	- KDOQI guidelines the Bangladeshi Food Composition Table. - International Phosphorous pyramid	- Adherence to specific cooking methods and vegetable preferences increased significantly. - Significant regression in serum potassium and phosphorus was reported after intervention.
Naseri-Salahshour et al., 2020 [29]	The effect of nutritional education program on quality of life and serum electrolytes levels in hemodialysis patients: A single-blind randomized controlled trial.	Iran	To ascertain the impact of a nutrition education program on the serum electrolyte levels and quality of life (QOL) of hemodialysis patients.	94	- Demographic questionnaire - Kidney Disease and Quality of Life™ Short Form (KDQOL-SF) - Medical records.	- Kidney Disease Outcomes Quality Initiative (NKF KDOQI) guidelines for nutrition in chronic renal failure	- After the establishment of the nutrition education program, the intervention group showed significant regression in serum potassium, sodium, phosphorus, and magnesium levels, and then no significant change was observed within the control group.
Chan et al., 2019 [30]	Multidisciplinary education approach to optimize phosphate control among hemodialysis patients.	Malaysia	To look into how well the program works for hemodialysis patients in terms of maintaining ideal phosphate control.	57	- Knowledge was assessed with a self-developed questionnaire - Medical records.	Not mentioned	- The dietary control part of the knowledge score increased significantly after the intervention.
Karavetian et al., 2016 [31]	Nutritional education for management of osteodystrophy: Impact on serum phosphorus, quality of life, and malnutrition	Lebanon	The purpose of the NEMO trial was to evaluate how intensive, tailored nutrition education from a specialist renal dietitian affected patients’ HRQOL and malnutrition inflammation syndrome.	394	- Questionnaires used in the study included: Quality of life, malnutrition assessment, and 24-h recall. - Medical records the comprehensive scoring system adapted from Kalantar-Zadeh et al. [12]. - HRQOL was measured using the Short-Form Health Survey-36.	- Transtheoretical model	- The length of hospital stay was lower than 3 days in all of the groups (DD, EP, and THD) - Daily protein intake (%) showed a significant regression (*p* < 0.05) in all groups, especially the DD group, which had the strongest regression. - The analysis of serum phosphorus (mmol/L) showed a significant decrease at T1 compared with T0 in the DD group, while no significant change occurred in the EP and THD groups. - Blood urea nitrogen decreased significantly at T1 in all groups but increased at T2 within acceptable limits.
Martins et al., 2017 [32]	EPIC Trial: education program impact on serum phosphorous control in CKD 5D patients on hemodialysis	Brazil	The Transtheoretical model of behavior change (TMBC) is used in this study to assess the effects of an NEP on hyperphosphatemia.	179	- Questionnaire - Medical records	- The transtheoretical model of behavior change	- After the nutrition education intervention, a significant reduction in serum phosphate levels was identified.
Liu et al., 2016 [33]	Use of a knowledge-attitude-behavior education program for Chinese adults undergoing maintenance hemodialysis: Randomized controlled trial	China	Evaluate the effects of a knowledge–attitude–behavior health education model on patients receiving maintenance hemodialysis in terms of their ability to learn about diseases and how to manage their health.	86	- Questionnaire - Parameters of chronic disease self-management.	- The knowledge–attitude–behavior education MODELE	- The intervention group showed a significant increase in Diet Principle knowledge (*p* < 0.001) Between baseline and 24 weeks. - The intervention group had more progress in self-management behavior scores in reasonable diet than the control group.
Oller et al., 2018 [34]	Clinical trial for the control of water intake of patients undergoing hemodialysis treatment	Brazil	To evaluate the effects of a motivational and educational intervention on patients receiving hemodialysis who have chronic kidney disease regarding their ability to control their fluid intake during the interdialytic phases.	192	- Instrument for the characterization of sociodemographic, economic, and clinical data.	- Motivation Bandura’s Theory	Regarding the variable Group (CG/IG), Intervention Group participants were 3.54 times more likely to reach the goal than CG.
Rizk et al., 2017 [35]	Cost-effectiveness of dedicated dietitians for hyperphosphatemia management among hemodialysis patients in Lebanon: results from the Nutrition Education for Management of Osteodystrophy trial	Lebanon	To evaluate the nutritional education provided by dedicated dietitians (DD) in terms of cost-effectiveness for the management of hyperphosphatemia in hemodialysis patients.	403	- The Short-Form (SF)-36 questionnaire QOL - The patients’ medical charts - Pilot-tested resource utilization questionnaire (cost measurement).	- The transtheoretical model of behavioral change	- The direct costs of nutrition education are incredibly low compared to the expenses of other interventions.
Bertonsello-Catto et al., 2019 [36]	Phosphorus Counting Table for the control of serum phosphorus levels without phosphate binders in hemodialysis patients.	Brazil	To assess the serum phosphorus levels in hemodialysis patients who do not use phosphate binder by monitoring measurements obtained before and following the intervention, using the Phosphorus Counting Table (PCT).	50	- Medical records.	Not mentioned	- After the intervention, a significant reduction in serum phosphate levels was noticed. - Serum PTH increased significantly in all patients, at the end.
Sevick et al., 2016 [37]	No Difference in Average Interdialytic Weight Gain Observed in a Randomized Trial with a Technology-Supported Behavioral Intervention to Reduce Dietary Sodium Intake in Adults Undergoing Maintenance Hemodialysis in the United States: Primary Outcomes of the BalanceWise Study.	USA	To assess the effectiveness of technology-based self-monitoring in conjunction with behavioral counseling for sodium restriction in hemodialysis (HD) patients.	160	- Medical records - Dietary recalls (face-to-face and via phone)	- Social cognitive theory (SCT)-based behavioral	The change in reported dietary sodium intake was significantly lower in the Intervention group at 8 weeks adjusted for diabetes status.
Chung et al., 2024 [38]	Adaptive nutrition intervention stabilizes serum phosphorus levels in hemodialysis patients: a multicenter decentralized clinical trial using real-world data	South Korea	This study aims to analyze the impact of an adaptive nutritional and educational approach on hemodialysis (HD) patients during their routine care, based on real information from electronic medical records.	153	- MIS, Korean-Beck Depression Inventory (K-BDI), - Short Form 36 (SF-36) - Self-Efficacy survey - Education satisfaction Likert scale survey - Electronic health record.	- The Korean Dietetic Association - The National Standard Table of Food Composition (version 9.1)	- The MIS (the malnutrition inflammatory score) results showed a lower score in the meal group than in the non-meal group (education only and control).
Torabi Khah et al., 2023 [39]	Comparing the effects of mHealth application based on micro-learning method and face-to-face training on treatment adherence and perception in hemodialysis patients: a randomized clinical trial.	Iran	Analysis of the impact of a mobile health app (mHealth) using the microlearning method versus face-to-face training on treatment adherence and perception among patients undergoing hemodialysis.	70	- End-Stage Renal Disease Adherence Questionnaire (ESRD-AQ).	- Clinical experience and observation of authors - The microlearning method	- Concerning adherence to fluid-intake limitation and dietary restrictions, the number of patients with full scores in these dimensions increased significantly immediately and 8 weeks after training via the Di Care app (*p* < 0.05).
Arad et al., 2021 [40]	Do the patient education program and nurse-led telephone follow-ups improve treatment adherence in hemodialysis patients? A randomized controlled trial.	Iran	This research aimed to analyze the impact of a patient education program and telephone follow-up by a nurse on treatment compliance in hemodialysis patients.	66	- Demographic questionnaire - The End-Stage Renal Disease Adherence Questionnaire (ESRD-AQ) - The laboratory results record sheet.	Not mentioned	- There was a significant increase in adherence to fluid restrictions during all four periods in the intervention group (*p* < 0.0005) compared with the control group (*p* = 0.126). - Adherence to dietary recommendations showed a significant increase during all four stages in the intervention group (*p* < 0.0005) compared with the control group (*p* = 0.344).
Huang et al., 2018 [41]	Effectiveness of self-management support in maintenance hemodialysis patients with hypertension: A pilot cluster randomized controlled trial.	China	This study aimed to assess the effectiveness of self-management support (SMS) in controlling blood pressure (BP) and health behaviors.	90	- Demographic and clinical characteristics modified balance formulas for salt intake.	- The self-management of chronic diseases	- Compared with baseline, patients in the self-management support group saw a significant reduction in salt intake, with a 1.7 g reduction at one month (*p* < 0.05), whereas there was no significant change in salt consumption in the Common intervention group (*p* > 0.05).
Jhamb et al., 2023 [42]	Effects of Technology-Assisted Stepped Collaborative Care Intervention to Improve Symptoms in Patients Undergoing Hemodialysis: The TĀCcare Randomized Clinical Trial	USA	To analyze the effectiveness of a stepped collaborative care approach versus attention control in decreasing fatigue, pain, and depression in ESKD patients undergoing prolonged hemodialysis.	160	- Demographic and clinical characteristics - Fluid restriction adherence (IDWG ≤ 3.5%).	Cognitive Behavioral Therapy	- No significant treatment effects in terms of fluid restriction adherence were identified between groups.
Wileman et al., 2016 [43]	Evidence of improved fluid management in patients receiving hemodialysis following a self-affirmation theory-based intervention: A randomized controlled trial.	UK	Analyze patients who received information about the dangers of inadequate fluid control versus patients who received information about their health risks after a brief self-affirmation activity.	89	- IDWG (kg)/questionnaires/Patient Information Sheet.	Self-affirmation theory/health information about fluid control, with kind permission to reprint from The American Association of Kidney Patients	- For self-affirmed patients, there was a considerable reduction in IDWG from baseline to six months with a mean reduction of 0.34 kg (*p* = 0.02). While in the intervention group, there was no reduction over the same period.
Valsaraj et al., 2021 [44]	Follow-Up Study on the Effect of Cognitive Behaviour Therapy on Haemodialysis Adherence: A randomized controlled trial.	India	This research aimed to analyze the impact of cognitive behavioral therapy (CBT) related to daily care on adherence to dialysis, fluids, medications, and diet in a sample of CKD patients undergoing hemodialysis.	67	- Patient records personal interviews - The adherence indicators.	Cognitive Behavioral Therapy	In comparison to the control group, the experimental group’s diet adherence ratings after six months increased significantly (*p* = 0.001) (+61.19 versus + 2.17).
Mateti et al., 2018 [45]	Impact of pharmaceutical care on clinical outcomes among hemodialysis patients: A multicenter randomized controlled study.	India	This research aims to analyze how pharmaceutical care influences medication adherence, hemoglobin (Hb) levels, blood pressure (BP), and interdialytic weight gain in hemodialysis patients.	153	- IDW = (Pre-HD weight − Post-HD weight).	WHO-FIP Pharmaceutical care model	- At different time intervals, the PCG demonstrated a statistically significant reduction (*p* < 0.001) in IDWG compared to the UCG.
Niznik et al., 2020 [46]	Time in Therapeutic Range for Dialysis Patients on Warfarin: Determination and the Effect of Dietary Intervention	USA	Measure the time spent in the therapeutic interval (TTR) of dialysis patients on warfarin and improve TTR by revising the diet and intervening in interacting foods.	15	- Demographic and clinical characteristics.	The institutional-approved patient education material	- After the intervention, the median percent time INR less than 1.5 was considerably lower (*p* = 0.042) than it was before (pre-intervention 10% versus post-intervention 8%).
De Fornasari et al., 2017 [47]	Replacing Phosphorus-Containing Food Additives with Foods Without Additives Reduces Phosphatemia in End-Stage Renal Disease Patients: A Randomized Clinical Trial	Brazil	This study aimed to confirm the effect of phosphatemia in patients with end-stage renal failure by substituting diets without additions containing phosphorus.	131	- Medical records - Anthropometric measurements - The interview.	The Brazilian Food Composition Tables	- 69.7% of the intervention group patients versus 18.5% in the control group (*p* < 0.001) fulfilled the required level of phosphorus in the serum. <5.5 mg/dL by the end of the study.
Howren et al., 2016 [48]	Effect of a Behavioral Self-Regulation Intervention on Patient Adherence to Fluid-Intake Restrictions in Hemodialysis: a Randomized Controlled Trial	USA	Using a sample of center hemodialysis patients with chronic kidney disease, a parallel-group randomized clinical study was conducted to assess the effectiveness of behavioral self-regulation intervention versus active control condition.	80	- Medical records.	Kanfer’s self-regulatory framework (24) of self-monitoring, self-evaluation, and self-reinforcement	- The intervention group’s interdialytic weight gain showed a slight within-subjects improvement, but despite this progress, they continued to be clinically nonadherent.
Wu et al., 2022 [49]	Effect of Self-Determination Theory on Knowledge, Treatment Adherence, and Self-Management of Patients with Maintenance Hemodialysis	China	This study aimed to look into how self-determination theory affected patients receiving maintenance hemodialysis (MHD) in terms of self-management, treatment compliance, and understanding of pertinent knowledge.	90	- The treatment compliance scale for maintenance hemodialysis patients with end-stage renal disease.	Self-determination theory	- Following the intervention, the intervention group’s scores for dietary compliance and fluid-intake compliance were significantly higher than the conventional group.
Pack et al., 2021 [50]	Randomized controlled trial of a smartphone application-based dietary self-management program on haemodialysis patients	South Korea	The purpose of this study was to create and evaluate the impact of a nutritional self-management program based on smartphone applications on the quality of life, self-efficacy, and biochemical markers of patients undergoing hemodialysis.	75	- The 15-item dietary self-efficacy questionnaire for hemodialysis patients, the 18-item Korean version of the Kidney Disease Quality-of-Life Instrument-Short Form (KDQOL-SF).	- The recommendations of a panel of hemodialysis experts, which consisted of two nephrology physicians, two nursing professors, and five hemodialysis nurses.	The application-based dietary self-management program was effective in lowering serum phosphorus and potassium in hemodialysis patients.
Mozafari et al., 2023 [51]	The effect of teach-back versus pictorial image educational methods on knowledge of renal dietary restrictions in elderly hemodialysis patients with low baseline health literacy	Iran	The purpose of this study was to compare the effects of teach-back and pictorial image educational techniques on elderly Iranian hemodialysis patients’ knowledge of renal dietary restrictions.	48	- Knowledge assessment questionnaire for hemodialysis (NKAQH) - Medical records.	Not mentioned	Following the intervention, the three groups’ mean knowledge scores increased, and the mean BUN score for the pictorial image group was found to have significantly decreased (*p* = 0.008).
Griva et al., 2019 [52]	The combined diabetes and renal control trial (C-DIRECT)—a feasibility randomised controlled trial to evaluate outcomes in multi-morbid patients with diabetes and on dialysis using a mixed methods approach	Singapore	This study looked into the acceptability and feasibility of the Combined Diabetes and Renal Control Trial (C-DIRECT) intervention in patients who had diabetes mellitus and end-stage renal disease (DM ESRD) at the same time.	42	- Dialysis Diet and Fluid Non-Adherence Questionnaire (DDFQ).	- Social Cognition Theory and Motivational Interviewing (MI) - The Elicit-Provide-Elicit framework	Following the intervention, no significant difference was reported in terms of the Dialysis Diet and Fluid Non-Adherence Questionnaire.

## Data Availability

Not applicable.

## Supplementary Materials

The following supporting information can be downloaded at https://www.mdpi.com/article/10.3390/healthcare12121197/s1, PRISMA 2020 Checklist; JBI Critical Appraisal Checklist for Case Series.
