# Supplementary material for: Impact of Nutrition Education on Various Health-Related Components of Hemodialysis Patients: A Systematic Review
Source: Healthcare (Basel). 2024 Jun 14;12(12):1197. doi: 10.3390/healthcare12121197 (PMC11203892; doi:10.3390/healthcare12121197)
Supplement: Supplementary file 1 [file healthcare-12-01197-s001.zip › healthcare-2955107-supplementary/JBI Critical Appraisal Checklist for Case Series.pdf]

JBI Critical Appraisal Checklist for Case Series										
	**A. L. Steiber et al (2015)**	**A. Valente et al (2022)**	**H. A. Fadlalmola et al(2020)**	**J. Yin et al(2021)**	***Niznik et al 2020***	**V. R. Bertonsello-Catto et al(2019)**	**C. T. B. Martins et al(2017)**	**M. W. Chan et al(2019)**	**T. Rahman et al(2022)**	**P. Vaz De Melo Ribeiro et al(2019)**
**Were there clear criteria for inclusion in the case series?**	YES	YES	YES	YES	YES	YES	YES	YES	YES	YES
**Was the condition measured in a standard, reliable way for all participants included in the case series?**	YES	YES	YES	YES	YES	YES	YES	YES	YES	YES
**Were valid methods used for the identification of the condition for all participants included in the case series?**	YES	YES	YES	YES	YES	YES	YES	YES	YES	YES
**Did the case series have consecutive inclusion of participants?**	YES	NO	NO	NO	NO	NO	NO	NO	NO	NO
**Did the case series have a complete inclusion of participants?**	NO	NO	NO	NO	NO	NO	NO	NO	NO	NO
**Was there clear reporting of the demographics of the participants in the study?**	YES	YES	YES	YES	YES	YES	YES	YES	YES	YES
**Was there clear reporting of clinical information of the participants?**	YES	YES	NO	YES	YES	YES	YES	YES	YES	YES
**Were the outcomes or follow-up results of cases clearly reported?**	YES	YES	YES	YES	YES	YES	YES	YES	YES	YES
**Was there clear reporting of the presenting site(s)/clinic(s) demographic information?**	YES	YES	NO	YES	YES	YES	YES	YES	YES	YES
**Was statistical analysis appropriate?**	YES	YES	YES	YES	YES	YES	YES	YES	YES	YES
**Total**	9	8	6	8	8	8	8	8	8	8

The Quantitative Study Quality Assessment Tool Rating	**Global rating for Paper**	**Detailed Rating For Paper**					
		**Selection Bias**	**Study Design**	**Confounders**	**Blinding**	**Data Collection Method**	**Withdrawals And Dropouts**
Howren et al.,2016	**WEAK**	Weak	Strong	Strong	Weak	Strong	moderate
Wu et al., 2022	**STRONG**	Moderate	Strong	Strong	Moderate	Strong	Strong
Pack et al.,2021	**MODERATE**	Strong	Strong	Strong	Weak	Strong	Strong
Mozafari et al.,2023	**STRONG**	Moderate	Strong	Strong	Moderate	Strong	Strong
Griva et al.,2019	**STRONG**	Strong	Strong	Strong	Moderate	Strong	Strong
Huang et al., 2018	**MODERATE**	Strong	Strong	Strong	Weak	Strong	Strong
Wileman et al.,2016	**WEAK**	Weak	Strong	Strong	Weak	Strong	moderate
Valsaraj et al., 2021	**STRONG**	Moderate	Strong	Strong	Moderate	Strong	Strong
Mateti et al., 2018	**MODERATE**	Strong	Strong	Strong	Strong	Weak	Strong
De Fornasari et al., 2017	**WEAK**	Strong	Strong	Weak	Weak	Strong	Strong

Jhamb et al., 2023	**MODERATE**	Strong	Strong	Moderate	Moderate	Strong	Weak
Torabi Khah et al., 2023	**MODERATE**	Weak	Strong	Strong	Moderate	Strong	Strong
Arad et al., 2021	**STRONG**	Strong	Strong	Strong	Moderate	Strong	Strong
Chung et al., 2024	**MODERATE**	Strong	Strong	Strong	Weak	Strong	Strong
**Griva et *al* (2018)**	**MODERATE**	Weak	Strong	Strong	Moderate	Strong	Moderate
**M. Karavetian *et al* (2015)**	**MODERATE**	Moderate	Strong	Strong	Weak	Strong	Moderate
**R. Rizk *et al* (2017)**	**MODERATE**	Strong	Strong	Strong	Weak	Strong	Moderate
**A. De Freitas *et al* (2020)**	**MODERATE**	Strong	Strong	Strong	Weak	Strong	Moderate
**B. Dsouza *et al (2023)***	**MODERATE**	Strong	Strong	Strong	Weak	Strong	Moderate
**J. J. Hernández Morante *et al* (2013)**	**MODERATE**	Moderate	Strong	Strong	Moderate	Strong	Weak
**D. E. St-Jules *et al (2022)***	**MODERATE**	Weak	Strong	Strong	Moderate	Strong	Strong
**J. Dawson *et al (2021)***	**MODERATE**	Weak	Strong	Strong	Moderate	Strong	Strong
**A. Nadri *et al*(2020)**	**MODERATE**	Moderate	Strong	Strong	Moderate	Strong	Weak
**H. Ebrahimi *et al* (2016)**	**WEAK**	Strong	Strong	Strong	Weak	Strong	Weak
**M. Karavetian *et al* (2013)**	**STRONG**	Moderate	Strong	Strong	Strong	Strong	Moderate
**V. Naseri-Salahshour *et* al (2020)**	**STRONG**	Strong	Strong	Strong	Moderate	Strong	Strong
**M. Karavetian *et al*(2016)**	**MODERATE**	Strong	Strong	Weak	Moderate	Strong	Moderate
**L. Liu *and al* (2016)**	**MODERATE**	Strong	Strong	Strong	Weak	Strong	Strong
**G. A. S. A. D. O. Oller *et al* (2018)**	**WEAK**	Moderate	Strong	Weak	Weak	Strong	Moderate
**R. Rizk *and al* (2017)**	**STRONG**	Moderate	Strong	Strong	Strong	Strong	Moderate
**M. A. Sevick *et al(2015)***	**STRONG**	Strong	Strong	Strong	Moderate	Strong	Strong
